# A Normotensive Patient with Primary Aldosteronism

**DOI:** 10.1155/2017/5159382

**Published:** 2017-04-02

**Authors:** Xiao Lin, Xiaoyu Miao, Pengli Zhu, Fan Lin

**Affiliations:** Department of VIP, Fujian Provincial Hospital, Fujian Medical University, 134 East Street, Fuzhou 350001, China

## Abstract

This study was to report a case of normotensive patient with primary aldosteronism who was admitted to our department recently. The patient was a 33-year-old male with right adrenal incidentaloma, but without any symptom. He has no history of hypertension, and blood pressure was normal when measured at multiple time points during hospitalization stay. The 24-hour ambulatory blood pressure prompted a normal blood pressure with the existence of circadian rhythm. The patient was diagnosed with primary aldosteronism by screening and confirmatory test. Due to the absence of symptom, surgery was not preferred. Blood pressure was found to be normal with the 2-month follow-up from discharge until now.

## 1. Introduction

Primary aldosteronism is a disease caused by increased aldosterone secreted in the zona glomerulosa of the adrenal cortex, with the symptoms of hypertension and hypokalemia as its main clinical manifestations. Although some patients with primary aldosteronism may not occur hypokalemia [1], but the vast majority of patients suffer from hypertension. Normotensive patients with primary aldosteronism are extremely rare. So far, only a total of 30 cases were reported in China and abroad [2, 3], and most of them were combined with hypokalemia. Recently, our department admitted a patient who was found with right adrenal incidentaloma by chest computed tomography (CT). Moreover, the patient was diagnosed with primary aldosteronism by screening and confirmatory test. He showed no symptom and sign, and no history and family history of hypertension, while the blood pressure and serum potassium were normal when measured at multiple time points during hospitalization stay. The case reported is shown as follows.

**Figure 1:**
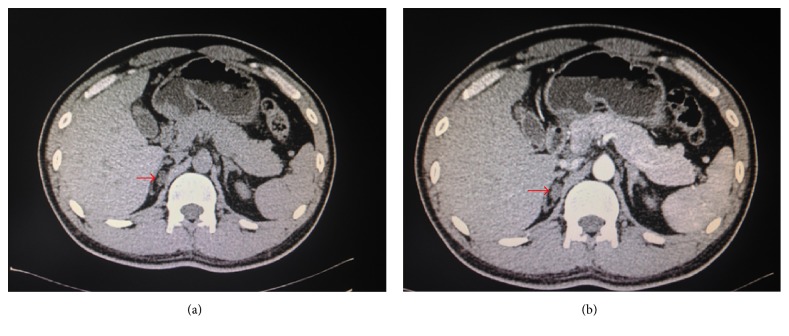
(a) Adrenal computed tomography scanning suggested right adrenal adenoma (pointed by red arrow). (b) Adrenal computed tomography enhancement indicated that the right adrenal adenoma was mildly intensified (pointed by red arrow).

**Figure 2:**
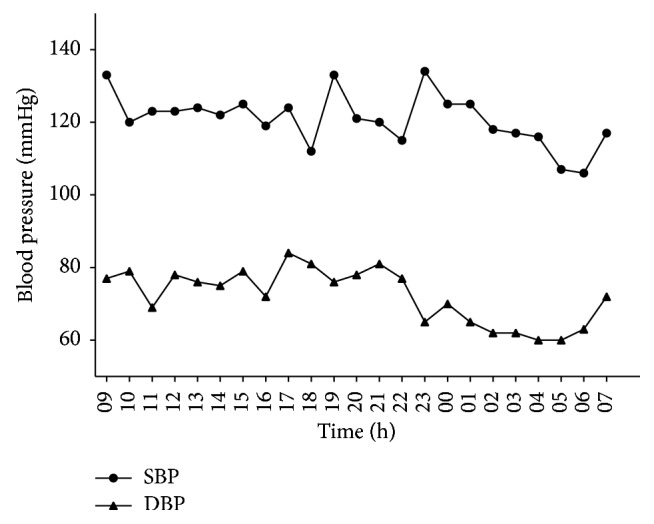
The trend of 24-hour ambulatory blood pressure of the patient.

The patient was a 33-year-old male admitted to the hospital due to the right adrenal incidentaloma found by chest CT on August 23, 2016. The patient had no symptoms, such as dizziness, headache, chest tightness, palpitations, fatigue, and periodic paralysis, as well as no history and family history of hypertension. Physical examination showed that blood pressure was 133/83 mmHg, while his height and weight were 180 cm and 65 kg, respectively, with the body mass index of 20.1 kg/m^2^. The patient was conscious and without palpable evidence of thyroid enlargement. Rales were not heard from the breath of the two lungs. Heart rate of the patient was 67 beats/min with regular rhythm, and no noise was heard from auscultation areas of each valve. The whole abdomen had no tenderness, without palpable evidence of liver or spleen enlargement. No edema was found in both lower extremities. Muscle force and muscular tension of all four limbs were normal symmetry, and reflexes of bilateral tendon were also normal. After admission, the three routine examinations including blood, urine, and feces were checked to be normal, while liver and kidney functions were normal as well. Blood potassium was 3.6–3.9 mmol/l (the normal range of 3.5–5.5 mmol/l) and serum sodium was 145 mmol/l (the normal range of 137–147 mmol/l). CT scanning of the mid abdomen plus enhanced scanning suggested that a low-density nodule similar to oval shape was found in the medial limb of the right adrenal gland, with the size of approximately 1.5 cm × 1.1 cm and CT value was −14 Hu. The lesion showed mildly enhancement (Figure 1). Related hormone test was performed to determine whether the right adrenal incidentaloma had function. 24-hour urinary vanilloid myristic acid was 37.00 umol, as well as serum adrenocorticotrophic hormone (08:00) of 55.52 pg/ml, serum cortisol (08:00) of 384.90 nmol/l, and serum cortisol (16:00) of 201.80 nmol/l, which were all in normal range. 24-hour urinary cortisol was 35.86 nmol (the normal range of 58.00–395.00 umol), which was a little bit low. The examinations of thyroid function, sex hormone, and growth hormone were all normal. Blood pressure of the right upper arm was measured at multiple time points during hospitalization stay, ranging between 116/60 mmHg and 133/88 mmHg. Blood pressures of the whole day by 24-hour ambulatory blood pressure monitoring were all less than 140/90 mmHg, averaged at 120/73 mmHg. The mean blood pressure at daytime was 120/75 mmHg, while it was 116/63 mmHg at night. The trend of blood pressure was dipper with double peaks and double troughs (Figure 2).

The ratio of plasma aldosterone (PAC) to plasma renin activity (PRA) was used as an indicator of screening for primary aldosteronism. The results indicated that PAC (in the supine position) was 246.21 ng/L (normal range was 59.50–173.90 ng/L) and PRA (in the supine position) was 0.10 Ug/lh (normal range was 0.05–0.79 Ug/lh), while the calculated ARR (in the supine position) was 246.21. PAC (in the erect position) was 228.33 ng/L (normal range was 65.20–295.70 ng/L), and PRA (in the erect position) was 0.35 Ug/lh (normal range was 0.93–6.56 Ug/lh), while the calculated ARR (in the erect position) was 65.24. ARRs of the patient in both supine position and erect position were greater than 50, suggesting that the possibility of primary aldosteronism was extremely great. The patient did not take any drug affecting the results of ARR and had a balanced sodium diet before screening test, as well as with normal serum potassium. According to the guidelines of the Endocrine Society of America [4], the saline infusion test was used to perform the confirmatory test of primary aldosteronism (Table 1). The results showed that PAC was 144.68 ng/L after saline infusion, which was greater than 100 ng/L, so it can be diagnosed as primary aldosteronism.

The patient had no symptom, and blood pressures were all normal when measured at multiple time points. During hospitalization stay, low serum potassium occurred once before saline infusion test. Subsequently, multiple times of the review of electrolyte demonstrated normal serum potassium. Therefore, drug therapy or surgical treatment was not performed. The patient conducted outpatient follow-up for 2 months from discharge until now without any symptoms found. Blood pressure of the right upper arm from family monitoring was 110–135/65–80 mmHg.

## 2. Discussion

The main clinical manifestation of primary aldosteronism is the combination with hypertension and hypokalemia. Although recent studies have found that the incidence of hypokalemia is only about 50% [5, 6] in patients with primary aldosteronism, the vast majority of patients suffer from hypertension. Based on this characteristic, the guidelines of the American Association of Endocrine [4] pointed out that indications of screening test of primary aldosteronism should include hypertension. However, there are few reports of normotensive patients with primary aldosteronism both in China and abroad. In 1972, Brooks et al. [7] first reported a normotensive patient with primary aldosteronism. To date, only a total of 30 such cases were reported in China and other countries. Rossi [8] analyzed the 26 normotensive patients with primary aldosteronism in the literature, showing that 85% of cases were from Europe and Asia, especially and mainly from Japan. Most of the patients were middle-aged and females. In addition, there were sporadic cases of familial hyperaldosteronism type I (FH-I) with normal blood pressure [2].

The patient in the current study is a young man, who was found to have right adrenal incidentaloma by chest CT. He had no history and family history of hypertension, as well as no symptoms of hypokalemia. During hospitalization stay, hypokalemia was found only once, and the remaining multiple time examinations showed normal plasma potassium. The patient was diagnosed by screening test of primary aldosteronism and confirmatory test. This finding posed a challenge to the current guidelines [4, 9] that only hypertension is used as the indicator of screening primary aldosteronism. For patient with adrenal incidentaloma who has normal blood pressure and without hypokalemia, primary aldosteronism cannot be easily excluded, which needs further screening of primary aldosteronism. From the literature, the sensitivity and specificity of saline infusion test are high. Rossi et al. performed saline infusion test for the 317 cases with ARR > 40, and, through analyzing sensitivity and specificity, 6.8 ng/dl (190 pmol/l) was selected to be the best critical value of aldosterone, with the sensitivity and specificity of 83% and 75% [10], respectively. Therefore, the case in the present study was diagnosed by this test.

For normotensive patient with primary aldosteronism, the mechanism of normal blood pressure is still unclear, which is considered to be associated with the following causes. ① Patients are still in the early stage of the disease. For example, the case reported in our study was without previous history of hypertension, while the multiple time blood pressures measured during hospitalization stay were all normal. Hypokalemia in this patient occurred only once. Markou et al. [11] reported that after 5 years of follow-up, normotensive patients with primary aldosteronism were more likely to develop hypertension than nonprimary aldosteronism patients. Therefore, as time goes by, hypertension and hypokalemia of this patient might gradually appear. ② Previous basal blood pressure of patients is low; even for the patients who have suffered from primary aldosteronism, it is still in normal range after increasing blood pressure [12]. ③ Vasodilator materials are in the body of patients, such as the increased secretion or sensitivity of prostaglandin E, kallikrein, and nitric oxide, while the sensitivity of vasoconstrictor substances is reduced [8]. ④ The aldosterone escape phenomenon occurs in the patients, and its mechanism could be the inhibition of endogenous renin-angiotensin-aldosterone system due to the role of genetic and environmental factors, thereby promoting sodium excretion and vasodilatation [2, 8]. ⑤ A long-term low-sodium diet may help the patient to maintain his blood pressure normal. ⑥ A minority of FH-I patients have normal or slightly increased blood pressure, but the mechanism is unclear.

Our case report has the following limitations. ① Because the absence of symptom was found, the patient did not have the will of surgery. Therefore, it lacked pathological evidence. Now, the follow-up is conducting for the patient in the study. ② FH-I is mainly unequal genetic recombination between 11*β*-hydroxylase CYP11B1 and aldosterone synthase CYP11B2 to form CYP11B chimeric gene. Therefore, it results that the aldosterone secretion of the patient is regulated by ACTH, and a minority of patients with FH-I will show a normal blood pressure. The patient in our study did not receive genetic examination. If necessary, genetic screening or the fludrocortisone suppression test should be considered for this patient, in spite of the modest specificity of the latter.

In summary, it is extremely rare that the patient suffered from primary aldosteronism is with normal blood pressure and without symptoms. We realize through this case that, for adrenal incidentaloma with normal blood pressure and serum potassium, it is necessary to conduct screening test to exclude the possibility of primary aldosteronism.

## Figures and Tables

**Table 1:** Comparison of the parameters before and after saline infusion test.

Parameters	Before	After
Serum potassium (mmol/L)	3.2	3.6
Serum sodium (mmol/L)	142	143
Serum cortisol (nmol/L)	451.70	295.10
Plasma aldosterone (ng/L)	179.93	144.68
Plasma renin activity (Ug/l·h)	0.22	0.01
Angiotensin (ng/L)	61.37	64.15
